# Peripheral Signals Mediate the Beneficial Effects of Gastric Surgery in Obesity

**DOI:** 10.1155/2015/560938

**Published:** 2015-04-15

**Authors:** Silvia Barja-Fernández, Cintia Folgueira, Cecilia Castelao, Rosaura Leis, Felipe F. Casanueva, Luisa M. Seoane

**Affiliations:** ^1^Grupo Fisiopatología Endocrina, Instituto de Investigación Sanitaria de Santiago de Compostela (IDIS), Complexo Hospitalario Universitario de Santiago (CHUS/SERGAS), Travesía Choupana s/n, 15706 Santiago de Compostela, Spain; ^2^Departamento de Pediatría, Universidad de Santiago de Compostela (USC), Instituto de Investigación Sanitaria de Santiago de Compostela, Complexo Hospitalario Universitario de Santiago (CHUS/SERGAS), Travesía Choupana s/n, 15706 Santiago de Compostela, Spain; ^3^CIBER Fisiopatología de la Obesidad y Nutrición, Instituto de Salud Carlos III, Spain; ^4^Departamento de Fisiología, Centro de Investigación en Medicina Molecular y Enfermedades Crónicas (CIMUS), Avenida Barcelona s/n, 15782 Santiago de Compostela, Spain; ^5^Laboratorio de Endocrinología Molecular y Celular, Universidad de Santiago de Compostela (USC), Instituto de Investigación Sanitaria de Santiago de Compostela (IDIS), Travesía Choupana s/n, 15706 Santiago de Compostela, Spain

## Abstract

Obesity is nowadays a public health problem both in the industrialized world and developing countries. The different treatments to fight against obesity are not very successful with the exception of gastric surgery. The mechanism behind the achievement of this procedure remains unclear although the modifications in the pattern of gastrointestinal hormones production appear to be responsible for the beneficial effect. The gastrointestinal tract has emerged in the last time as an endocrine organ in charge of response to the different stimulus related to nutritional status by the modulation of more than 30 signals acting at central level to modulate food intake and body weight. The production of some of these gastric derived signals has been proved to be altered in obesity (ghrelin, CCK, and GLP-1). In fact, bariatric surgery modifies the production of both gastrointestinal and adipose tissue peripheral signals beyond the gut microbiota composition. Through this paper the main peripheral signals altered in obesity will be reviewed together with their modifications after bariatric surgery.

## 1. Introduction

Overweight and obesity are serious public health problems in the developed and developing world. It has been estimated that at least 3.4 million people die each year as a result of both disorders [1]. In fact, obesity is considered as an epidemic of the 21st century by the World Health Organization (WHO) due to continued increase over the past decades in the prevalence of this disorder. In the European Union, obesity affects 10–30% of adults [2] whereas in the USA between 30 and 35% of the general population are obese [3]. Child and youth obesity are particular concern since the National Institutes of WHO has reported that more than 40 million children under the age of 5 years were overweight or obese in 2012 [4]. Children with overweight typically progress to become obese adolescents and adults [5]. According to WHO, more than 700 million adults and children will be obese in 2015 [6].

Obesity is defined as abnormal and excessive fat accumulation caused by an imbalance between energy intake and caloric expenditure. This disorder results from the increase of high caloric food intake and reducing physical activity. Moreover, in developing countries malnutrition and nutrient deficiencies coexist with an industrialized profile of lifestyle, characterized by the high energy food ingestion and low physical activity [7, 8].

Both overweight and obesity are associated with a very high prevalence of comorbidities, emphasizing metabolic syndrome, hypertension, type 2 diabetes (TD2), dyslipidemia, coronary heart disease, and certain cancers (colon, endometrial, prostate, and breast). Moreover, obesity is also associated with sleep apnea [9], psychological disorders, and pregnancy complications [10]. It was estimated that 1 kg of weight gain increases the diabetes risk by 4.5–9% and cardiovascular risk by 3.1% [11].

## 2. Energy Balance Regulation

Over millions of years human ancestors lived under selective pressure characterized by hostile environment and intense physical activity. It was postulated that to ensure survival under these situations it was necessary to develop a thirty genotype which enable individuals to efficiently select genes responsible for storage fat reserves during periods of food abundance to be used as a source of energy during periods of food shortage [12]. The body weight and appetite control is a complex and interactive mechanism regulated by nervous, hormonal, and metabolic pathways.

### 2.1. Central Regulation of Energy Balance

The regulation of food intake and energy expenditure at the central nervous system (CNS) involves the action of neurotransmitters and neuromodulators. The hypothalamus, specially the hypothalamic arcuate nucleus (ARC), is the central brain structure responsible for food intake regulation. At this level, neurons coexist expressing anorexigenic peptides such as proopiomelanocortin (POMC) and cocaine and amphetamine-regulated transcript (CART), with an adjacent set of neurons coexpressing orexigenic peptides such as agouti-related peptides (AgRP) and neuropeptide Y (NPY) [13]. Hypothalamic neurons respond to peripheral signals (as leptin, ghrelin, glucose, and insulin) and the main neurotransmitters by modifying the orexigenic/anorexigenic neuropeptides production to finally regulate food intake. In addition, recent researches in central regulation of energy homeostasis field have reported that hypothalamic lipid metabolism is a key mechanism regulating energy homeostasis [13]. In fact, the peripheral signals involved in energy balance regulation (leptin, ghrelin, and cannabinoids) modulate the main lipid metabolism enzymes such as MAP-activated protein kinase (AMPK) and acetil-CoA carboxylase (ACC) [14].

It has been proposed that the endocannabinoid (EC) system is another central target for energy balance regulation. It has been reported that cannabinoid receptor type 1 (CB1) agonism ensures palatable food consumption, while the antagonism for CB1 induces weight loss in obesity status possibly improving leptin sensitivity at central level [15].

### 2.2. Peripheral Regulation of Energy Balance

In the last time, the classical idea of an only central regulation of energy balance has changed to a novel point of view that considers the regulation of energy balance as the result of a complex interaction between brain and peripheral organs. In this sense, the stomach-brain communication, regulated under neural and hormonal control, allows stomach to play a role in the homeostatic mechanism participating in body weight maintenance. The neural stomach-brain mechanism, associated with central functions such as appetite and reward [16], is exerted by the autonomic nervous system (ANS) innervating the stomach with a leading role of the vagus nerve [17]. The afferent fibers of the vagus reach the dorsal brainstem and from here to different brain centers, as the hypothalamus, to modulate both orexigenic and anorexigenic signals in charge of regulating energy balance [16]. In addition, the enteric nervous system comprises a set of neurons localized in gastric myenteric and submucous plexus required for different gastrointestinal functions as secretion [18] but also primarily involved in responding to nutrient signalling [19].

#### 2.2.1. Gastrointestinal Tract Derived Signals Involved in Food Intake Regulation

The gastrointestinal tract is the largest endocrine organ in the body and gastrointestinal-derived signals are crucial for energy balance regulation. It has described that there exist more than thirty peptides secreted from enteroendocrine cells of the gastrointestinal tract in response to food intake. These peptides participate in hunger/satiety perception by interacting with hormones involved in body weight regulation to maintain energy homeostasis [20]. The majority of satiation-inducing peptides mediate their effects in CNS via vagal afferent fibers, although some exert their effects directly reaching the hypothalamus from the circulation [21].

*The EC System*. The EC System is not only present at central level. The major peripheral organs involved in metabolism regulation are targets for cannabinoid actions. In fact, the EC system participates in lipogenesis, glucose homeostasis, and insulin sensitivity [22]. Recently, it was showed that CB1 receptors are expressed in the stomach, in the same neuroendocrine cells producing ghrelin [23].

*Ghrelin*. Ghrelin, an endogenous ligand for the growth hormone secretagogue (GHS) receptor, is expressed mainly in the neuroendocrine cells from the gastric fundus [24, 25]. Ghrelin isolation from the stomach highlighted the emerging role of the stomach as an endocrine organ. In addition, it is also expressed in the entire gastrointestinal tract and other tissues such as hypothalamus, pituitary, testis, ovary, placenta, and heart [26]. The fact that ghrelin-circulating concentrations decreased by 65% after gastrectomy, both in humans and rodents, suggests that the stomach is the main source of this hormone in the organism [27]. The primary function of ghrelin is to regulate appetite acting as an orexigenic signal, being the first gastric derived peptide with appetite stimulating proprieties [24, 28, 29]. Additional ghrelin effects include gastric acid secretion and gastric motility [30]. Ghrelin constitutes a crucial link between the stomach and brain to regulate energy homeostasis by acting at the NPY/AgRP pathway in the hypothalamus [21, 28]. Nutritional status regulates ghrelin production from the stomach and circulating levels. Elevated plasma levels in fasting condition were described as a consequence of an increase in ghrelin secretion from the stomach as demonstrated by the use of a novel gastric tissue explants model [31]. In addition, it has been reported that the exposure to sensory stimuli related with the food (without true feeding) modifies gastric ghrelin secretion and its plasma levels in the same way as real intake [31]. Therefore, relevant factors involved in this process are the CNS sensorial stimuli and they emphasize the relevance of brain-stomach connection in energy balance regulation.

Ghrelin-circulating levels in obesity are low and are negatively correlated with body mass index (BMI), percent body fat, and leptin levels [32]. It is thought that this correlation is due to a physiological adaptation to long-term positive energy balance [33].

*Cholecystokinin (CCK)*. CCK is released into circulation from type I endocrine cells present in the duodenum and jejunum mucosa immediately after nutrients intake, and especially to lipids and proteins ingestion. CCK acts as an anorexigenic hormone as showed by its food intake inhibitory effects [34]. Once released in response to food intake, it exerts the anorexigenic effect through the activation of the CCK1 receptor in the afferent vagal nerves on the gastrointestinal tract [35]. Moreover, CCK interacts with other metabolic signals such as leptin, ghrelin, and peptide YY in order to regulate energy balance [36].

In obese individuals a dysfunction in both basal and stimulated by fatty meal CCK plasma levels has been reported [37].

*Glucagon-Like Peptide 1 (GLP1)*. GLP1 is an anorexigenic hormone encoded by preproglucagon gene whose posttranslational processing generates different peptides products (GLP-1, GLP-2, glucagon, glicentin, and oxyntomodulin) depending on the site expression. GLP-1 is mainly synthesized and secreted by enteroendocrine L cell located in the duodenum and small and large intestine. It is also present in the pancreas and hypothalamus [38, 39]. GLP-1 secretion in the gastrointestinal tract is regulated by glucose and fatty acid levels after food intake and vagus nerve stimulation [40, 41]. This gut hormone is considered an incretin since several experimental studies, both in animal and human models, have showed that the main GLP-1 action is reducing circulating glucose by stimulating insulin production and secretion from pancreatic *β*-cells and inhibiting glucagon secretion [42, 43]. This glucose-lowering agent activates its own GLP-1 receptor also in the CNS producing central effects as decelerating the rate of gastric emptying and gut motility, suppressing appetite, and reducing body weight [42, 44, 45]. Several rodent studies have showed that GLP-1 or analogs of GLP-1 administration produce a dose-dependent inhibition of food intake attributed to decrease both in meal size and in frequency [46–50]. In humans, the clinical data has showed that this peptide reduces appetite, hunger, and food consumption and promotes satiety [51–55].

With the fact that GLP-1 acts as an inhibitor of food intake, one would expect a decrease in circulating GLP-1 levels in obesity status. However, conflicting results were reported. Initially a hypersecretion was found [56], but in other studies normal [57] or reduced GLP-1 plasma levels were described in obese subjects [58–61]. Moreover, GLP-1 secretory responses were decreased in diabetic subjects [62, 63]. In addition, in obese diabetic patients GLP-1 levels were negatively correlated with BMI [64].

*Nesfatin-1*. Nesfatin-1 is a peptide derived from the precursor nucleobinding protein 2 (NUCB2), identified in 2006 [65] as a satiety factor. At central level, NUCB2 is coexpressed with appetite-regulatory peptides (such as NPY and CART) in hypothalamic nucleus which are involved in food intake control [66]. At peripheral level, nesfatin-1 expression was reported in stomach, pancreas, heart, testis, and adipose tissue [67]. In the stomach, nesfatin-1 expression was showed in the same cells producing ghrelin [68]. Nesfatin-1 is a novel anorexigenic modulator of food intake and body weight by its actions in reducing food intake, weight gain, and fat depots [65, 67]. With respect to nesfatin-1 production, a regulatory role for nesfatin-1 on gastric motor activity as the mediator of its anorexigenic actions has been suggested [65, 66, 69]. The fact that in the stomach nesfatin-1 expression takes place in the same cells producing ghrelin at a tenfold higher level than in the brain and that the vagus nerve is implicated in nesfatin-1 anorexigenic effect suggests that, as with ghrelin, the main source in the organism for nesfatin-1 is the stomach. Moreover, taking into account the opposite effects of both peptides on food intake, adiposity, and body weight, the possibility of an interaction between nesfatin-1 and ghrelin to regulate energy homeostasis should be considered.

Different studies have showed that both gastric expression of NUCB2/nesfatin-1 and circulating levels are increased in obese subject with respect to lean individuals levels [67, 70, 71]. Moreover, plasma nesfatina-1 concentration was positively correlated with BMI [70, 71].

*Gastrin*. Gastrin is a gastrointestinal peptide produced and released mainly by the G-cells in the antral and duodenal mucosa, although its expression was also described in the pancreas. The main biological effects of this hormone are stimulation of acid secretion from gastric parietal cells and stimulation of mucosal growth in the acid-secreting part of the stomach [37, 72]. Moreover, it has been postulated that gastrin presents an insulinotropic effect [73].

It has been reported that EC system in the digestive tract mediates gastric acid secretion among other actions such as gastric empting and contractility [74, 75]. The cannabinoid agonists inhibit pentagastrin-evoked acid secretion. It has been showed in rat's studies that the mechanism underlying the antisecretory effect of EC is mediated by CB1 receptors in an agonist's dose-dependent manner [74, 75]. In addition, this antisecretory effect is only effective when the CB-1 receptor agonists are administrated peripherally [76].

#### 2.2.2. Main Adipokines Involved in Energy Balance Regulation

Recently, an increasing number of adipose tissue derived signals named adipokines have shown to regulate energy balance by communicating to the brain the organism energy status. In the past, adipose tissue was only considered as a lipid reservoir of the whole body. Nevertheless, this classical vision was banished when leptin was isolated in 1999. This event supposes an important starting point in the study of the adipose tissue as an endocrine organ.

*Leptin*. Leptin is a protein hormone produced and secreted mainly by white adipose tissue (WAT) and plays an important role in the regulation of food intake (satiety and appetite) and energy expenditure [77]. Their circulating levels correlate positively with the fat mass. Leptin key function is regulating appetite acting through the leptin receptors in the arcuate nucleus of the hypothalamus. The mechanism of leptin action involves the inhibition of orexigenic neuropeptides in parallel with increase in anorexigenic peptides, decreasing hunger [78].

In obese subjects, leptin-circulating levels are increased and do not reduce the food intake [77]. In addition, exogenous leptin administration in these patients does not influence neither appetite nor body weight due to central leptin resistance existence.

*Adiponectin*. Adiponectin is synthesized and secreted mainly by adipocytes into circulation. At central level, adiponectin acts in the brain to produce an increase in energy expenditure and weight loss [79]. One of the key roles of adiponectin is acting as an endogenous insulin sensitizer [80] but also it exerts anti-inflammatory and antiapoptotic actions on different cell types [79].

Adiponectin levels are downregulated under adverse metabolic conditions, as adverse fat distribution and adipose tissue dysfunction typical of obesity status, resulting in decreased adiponectin plasma levels [79]. Accordingly, adiponectin-circulating levels correlated inversely with body weight and especially with visceral fat mass [81].

Besides being considered a gastrokine,* Nesfatin-1* is also categorized as an adipokine due to being synthesized in adipose tissue [82].

#### 2.2.3. Gut Microbiota Role in Energy Balance Regulation

A novel key “organ” proposed to be involved in energy homeostasis is the gut microbiota. This is an environmental factor evolving with organism from birth and dietary habits. Besides participating in different intestinal functions (as defense against pathogens and immunity among others), several studies have reported that gut microbiota is involved in the fat mass development and altered energy homeostasis, regulating fat storage [83, 84]. In pathological conditions such as obesity and TD2 the microbiota is able to control the host metabolism and participate in development of low-grade inflammation [85–87].

Obesity pathology is associated with changes in the gut microbiota composition and diversity, which gut permeability alters. Several researches have showed important changes in two gut microbiota-dominant phyla: Firmicutes and Bacteroidetes. In obesity animal models an increase in Firmicutes and decrease in Bacteroidetes were reported [88–91]. In humans, the obesity pathology is also associated with changes in principal bacterial phyla abundance. Generally, the Firmicutes/Bacteroidetes ratio is increased in obese subjects [92, 93].

Recently, a new pathophysiological mechanism linking to gut microbiota, EC system, and adipogenesis has been reported. Gut microbiota alterations activate the gut EC system increasing gut permeability, which produce endotoxemia, exacerbate gut barrier disruption, and activate peripheral EC system, in both intestine and adipose tissue. Under obesity pathological conditions, the endotoxemia and EC system activation dysregulate adipogenesis [94].

## 3. Obesity Treatments

The scientific concern about current alarming obesity rates, in industrialized as in developing countries, has showed that the studies have been focused on understanding the main physiologies mechanisms controlling energy homeostasis to develop efficacy obesity treatments. Currently, there are three strategies for treatment of obesity: lifestyle interventions, pharmacotherapy, and bariatric surgery [95]; the main goal of these strategies is weight loss. Lifestyle modifications in the patterns of diet and increase in daily exercise can be useful in obesity prevention; however in most cases these procedures only achieve short weight loss [96, 97]. Pharmacological interventions have shown limited success and currently antiobesity drugs options are limited. However, new drugs acting through the CNS pathways and/or peripheral adipose tissue and gastrointestinal tract signals are under research and clinical development [82, 98–104]. In fact, FDA has just approved two novel drugs in September 2014 [105].

The third obesity treatment tier is bariatric surgery. Surgical candidates are patients with BMI ≥ 40 kg/m^2^ or with a BMI ≥ 35 kg/m^2^ who have associated high-risk comorbid conditions (cardiopulmonary disease or TD2) [106]. The clinical benefits of bariatric surgery in achieving weight loss and improving metabolic comorbidities have largely been attributes to changes in the physiological responses of gastrointestinal hormones and adipose tissue metabolism [107, 108]. Moreover, the role of bariatric surgery in the prevention and treatment of TD2 has garnered attention, since it has been demonstrated that it markedly reduced the incidence of TD2 developing by 78% [109, 110]. At present, the most effective obesity treatment, in terms of weight loss, comorbidity reduction, and enhanced survival, is bariatric surgery [21, 111]. However, owing to concerns about perioperative mortality, surgical complications, and the frequent need for reoperation, this procedure tends to be reserved for the morbidly obese [112].

### 3.1. Bariatric Surgery

Weight loss surgery is divided into the following three types: restrictive, metabolic, and restrictive/metabolic. The first one includes the adjustable gastric band (AGB), which encircles the stomach cardia with an inflatable silicone ring, and the sleeve gastrectomy (SG), which removes most of the stomach greater curvature. Both procedures limit the patient rate of food intake. Metabolic procedures include duodenal-jejunal bypass (DJB) and biliary-pancreatic diversion (BPD). DJB procedure consists in duodenum separation from the stomach outlet connecting a jejunal branch in its place. In this situation, nutrients bypass the duodenum, which is exposed to undiluted bile, and nutrients and bile mixture taking place in the jejunum. BPD method involved a much longer intestinal bypass than DJB, in which the alimentary limb is anastomosed to the ileum. The roux-en-Y gastric bypass (RYGB) is considered as both restrictive and metabolic weight loss surgery. In this intervention the stomach is separated into a small upper pouch which is anastomosed to a roux jejunal limb. The remnant stomach remains attached to the biliary limb, into which the bile drains. In this way foods have earlier contact with the mid and distal small bowel.

Each weight loss surgery has its pros and cons. BPD can lead to severe nutrient deficiencies, making the SG or RYGB be the procedure selected [113, 114]. It has been reported that RYGB induces greater weight loss and resolution of diabetes than other procedures. For example, RYGB surgery has greater efficacy for weight loss in obese individuals than AGB (25% weight loss versus 14%) [115]. This indicates that the specific gut anatomical manipulations may produce different physiological effects [116].

## 4. Changes after Bariatric Surgery

**Figure 1:**
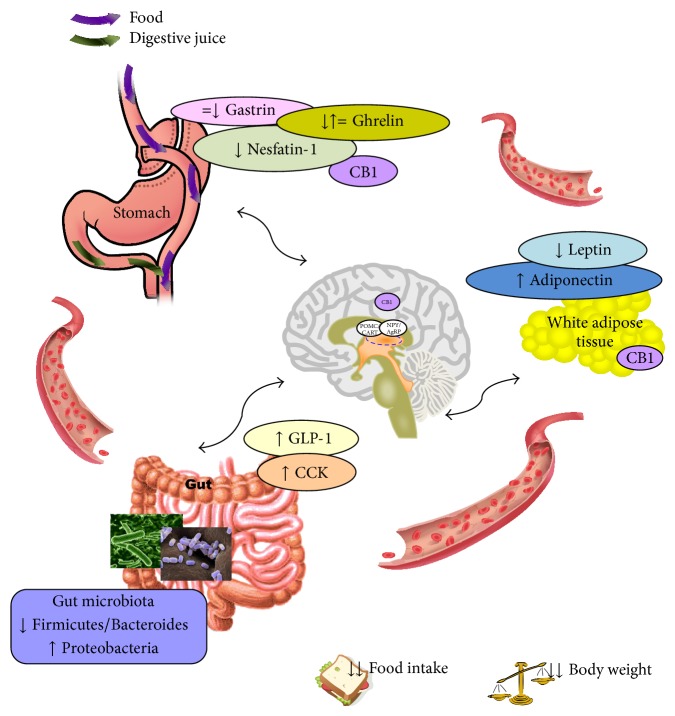
The figure represents the major organs involved in the modulation of peripheral signals (ghrelin, nesfatin-1, gastrin, leptin, adiponectin, GLP-1, CCK, and gut microbiota) after Roux-en-Y gastric bypass (RYGB) surgery. The increase/decrease in the signals is designed by arrows. CB1: cannabinoid receptor type 1; CCK: cholecystokinin; GLP-1: glucagon-like peptide 1.

At present, bariatric surgery is the most effective strategy to treat obesity due to the clinical benefits in weight loss achieved and improving metabolic comorbidities [95]. Between the different procedures for bariatric surgery, the RYGB is currently the key effective bariatric surgery procedure for long-term weight loss maintenance [117, 118]; therefore the metabolic changes after bariatric surgery described in this review mainly focus on this weight loss surgery (Figure 1).

Compare with other gastric surgery procedures RYGB surgery induces greater and sustain rapidly weight loss, between 65 and 75% of excess body weight and fat mass [119]. Moreover, after RYGB improvement of glucose homeostasis and dyslipidemia and decreased diabetes it has been reported [110, 119, 120].

Taking into account the relevant role of RYGB in improving obesity and associated pathologies, an increasing number of studies focus on the main modifications of peripheral signals regulating energy balance after RYGB.

### 4.1. Hormonal Changes Associated with Gastric Surgery

Studies of ghrelin-circulating levels after bariatric surgery showed different controversial results with the surgery procedure. With respect to RYGB strategy, studies have showed that ghrelin levels decreased, increased, or did not change [121–126]. These different results found on ghrelin plasma levels after RYGB can be explained by the variability in the time at which blood was drawn, the operative technique used, and the remnant gastric pouch in any case. Regarding the remaining bariatric surgery procedures the ghrelin levels are variables. For example, several studies have reported an increase or no change in ghrelin levels after AGB [127–131], a decrease after SG procedure [124, 132, 133], and a decrease or no change both in DJB and in BPD [133, 134].

The bibliography reported controversial data about the modification of the pattern of secretion of CCK after gastric surgery. In fact, the published works about this topic have revealed great variations in the production of CCK depending on the type of procedure. The studies performed after jejunoileal bypass surgery showed an increase in the density of CCK mucosal enteroendocrine cells [135] both in humans and in rats [136]. Moreover, in patients after this surgery procedure CCK levels were increased 3 months [137] and even 20 years [138] after surgery. The first studies with RYGB surgery failed to find modifications in the postprandial CCK response to an enriched protein-fat mail six months after surgery [139]. However, more recently, changes in postprandial CCK levels were reported in patients 2 weeks after surgery [140]. All together might lead to proposing that the increase in postprandial CCK levels might contribute to early satiety after RYGB surgery.

Different studies have showed that circulating GLP-1 levels increase after RYGB surgery, prior to weight loss occurrence which may participate in remission of diabetes [122, 130, 141–143]. In the other bariatric surgery procedures variable responses to circulating GLP-1 levels were showed [134].

It has been demonstrated that circulating nesfatin-1 levels decreased after RYGB surgery [144].

Several studies in animals and humans have revealed that the changes in circulating levels of gastrin after surgery depend on the selected procedure. It has been reported that the levels of serum gastrin strongly increase at 6 and 36 weeks after SG surgery in rats. However, this hypergastrinemia found after SG was not observed in the case of DJB procedure [145]. Similar results were found in patients who showed high circulating gastrin levels after SG, but no modifications were observed after AGB [146, 147]. Regarding RYGB procedure, fasting plasma concentrations of gastrin were unchanged after surgery, although under postprandial conditions these levels were decreased [140]. The fact that SG surgery produces an increase in circulating gastrin levels can be caused by the major reduction of the oxyntic mucosa achieved by this procedure. Accordingly, this reduction of the mucosa leads to hypoacidity decreasing the inhibitory effect of luminal hydrochloric acid on gastrin secretion [145, 148].

Several studies have reported a decrease in circulating leptin levels after RYGB intervention [123, 126, 130, 149, 150]. This fact is not striking taking into account the fact that the circulating leptin concentrations are correlated with body fat mass percent and it has been proved that RYGB surgery reduces adiposity. AGB procedure is the only bariatric surgery strategy where an increase in leptin levels has been reported [130, 151–153], although there are studies which showed no changes or decrease in post-AGB leptin levels [134].

The studies that have researched the adiponectin levels after bariatric surgery have reported an increase in circulating levels after RYGB procedure [142, 150, 154–158].

### 4.2. Gut Microbiota Changes

Several studies show that gut microbial ecology changes significantly after bariatric surgery. Recently, it has been reported that RYGB procedure modifies the gut microbiota profiles in both rats and humans [93, 159, 160]. Gut microbiota analysis has reported that a decrease in the Firmicutes/Bacteroides ratio and an increase in Proteobacteria taxonomic group are induced after RYGB [93, 117, 160–163]. Liou and coworkers have showed that these marked modifications in gut microbiota are due to gastrointestinal reconfiguration caused after RYGB intervention [162]. In the case of Proteobacteria group, which is a bile-tolerant bacterium, the greater amount of free bile acids released into the intestine after RYGB create an environment more hospitable which is responsible for Proteobacteria growth [134]. Moreover, it has been demonstrated that RYGB-associated gut microbiota is able to produce a decrease in body weight and adiposity, probably reducing the ability to harvest energy from diet or regulating lipid metabolism and upregulating energy expenditure through microbial products [93, 162].

## 5. Conclusions

The main mechanism responsible for the beneficial effect of bariatric surgery could be related to variations in the pattern of secretion of peripheral signals, especially those derived from the gastrointestinal tract. However, until now controversial results have been published which do not allow fully elucidating the mechanism responsible for the restoration of body weight after bariatric surgery.
